# Early-onset colorectal cancer incidence in Norway: a national registry-based study (1993-2022) analyzing subsite and morphology trends

**DOI:** 10.1016/j.esmogo.2024.100065

**Published:** 2024-08-02

**Authors:** M.F. Ystgaard, T.Å. Myklebust, J. Smeby, I.K. Larsen, T.K. Guren, E.H. Kure, K.M. Tveit, B. Glimelius, M.G. Guren, J. Hamfjord

**Affiliations:** 1 Department of Oncology, Oslo University Hospital, Oslo; 2 Department of Registration, Cancer Registry of Norway, Norwegian Institute of Public Health, Oslo; 3 Department of Research and Innovation, Møre and Romsdal Hospital Trust, Ålesund; 4 Department of Cancer Genetics, Institute for Cancer Research, Oslo University Hospital, Oslo; 5 Division of Cancer Medicine, Institute of Clinical Medicine, Faculty of Medicine, University of Oslo, Oslo, Norway; 6 Department of Immunology, Genetics and Pathology, Uppsala university, Uppsala, Sweden; 7 Lovisenberg Diaconal University College, Oslo, Norway

**Keywords:** early-onset, incidence, colorectal cancer, primary tumor location, neuroendocrine, adenocarcinoma

## Abstract

**Background:**

The overall incidence of colorectal cancer (CRC) has decreased or stabilized in most high-income countries, but an increase is observed in adults <50 years of age, termed early-onset colorectal cancer (EOCRC).

**Materials and methods:**

We conducted a nationwide registry-based study to provide up-to-date incidence patterns of EOCRC, with an emphasis on age-specific differences in tumor site and morphology. We extracted data from the Cancer Registry of Norway, with completeness estimated to be >99%. We calculated age-standardized incidence rates and used joinpoint regression to provide annual percentage change (APC) and average annual percentage change (AAPC). National screening started in Norway in 2022; hence, this study is on a largely screening-naive population.

**Results:**

There were 107 523 cases of CRC diagnosed between 1993 and 2022 eligible for analysis. The incidence of EOCRC (20-49 years) increased by 66% (AAPC 1.3), caused by left-sided colon and rectal cancers. The age group 50-74 years had a stable overall incidence (AAPC –0.2). In the age group ≥75 years, an increased incidence (AAPC 0.8) was observed, mainly by right-sided colon cancer, which increased until 2016 (APC 2.5), followed by a decrease (APC –0.9). EOCRC adenocarcinomas increased by 77% (AAPC 1.4). Neuroendocrine neoplasms were rare (1.4%), but increased in all age groups (AAPC 4.2). EOCRC showed a significantly higher proportion of distant metastases at time of diagnosis compared with the age group ≥75 years (28.6% versus 18.2%, *P* < 0.001).

**Conclusions:**

We observed a sustained increase in EOCRC, caused by left-sided colon and rectal cancers. Unlike countries with established screening programs, the incidence of the age group >50 years remained stable or increased, but declined in recent years.

## Highlights

• EOCRC is increasing, mainly caused by left-sided colon and rectal cancer.
• Adenocarcinomas increased by 77% (AAPC 1.4) in the age group 20-49 years.
• In the older age group (≥75 years) right-sided colon cancer has increased, but is now declining.

## Graphical abstract

**Figure:**
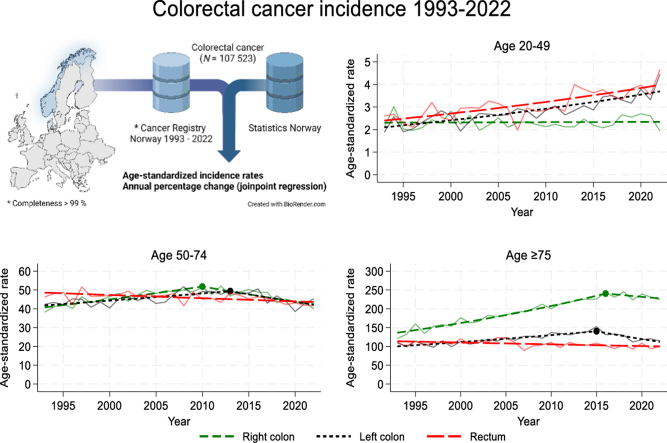

## Introduction

Colorectal cancer (CRC) is one of the most common malignancies worldwide and is the second leading cause of cancer death.^1^ Norway has a high incidence of CRC, which has been increasing until the past two decades, when it has subsequently leveled out.^2^^,^^3^ The incidence in most high-income countries has been either stable or decreasing, but there have been several reports of increasing incidence rates in younger adults (<50 years old), termed early-onset colorectal cancer (EOCRC).^4^^,^^5^

Compared with late-onset CRC (LOCRC, age ≥50 years), EOCRC is more likely to occur in the left colon or the rectum and is more frequently diagnosed in later stages.^6^ Previous studies have shown a higher rate of mucinous and signet ring cell differentiation, and of poorly or undifferentiated carcinomas.^7^^,^^8^ However, when excluding mismatch repair-deficient tumors, a recent study found no major differences in histopathological or genetic characteristics between EOCRC and LOCRC.^9^ Although there is a higher proportion of hereditary cancers among EOCRC, most cases (75%-84%) are sporadic.^5^

Anatomical subsite is a prognostic factor for stage IV CRC and predictive for the response to treatment.^10^ There are different carcinogenetic pathways leading to cancers, with varying molecular features corresponding to CRC subsites.^11^, ^12^, ^13^ We have previously shown in a nationwide cohort study that left-sided colon cancer has a more favorable prognosis than right-sided colon cancer, with the prognostic impact gradually increasing over the past four decades.^14^ Different molecular features related to the subsite may imply different etiologies that could be of importance for prevention strategies.

Neuroendocrine neoplasms (NENs) in the colon or rectum are commonly classified as CRC in cancer registries. The incidence of colorectal NENs has increased in the past decades.^15^^,^^16^ Montminy et al.^17^ found that NENs accounted for an increasing proportion of EOCRC cases in the United States, up to 22% of cases in some subgroups.

In 2022, Norway introduced a national screening program for CRC, starting at age 55 years. Our study aims to analyze the incidence of CRC in Norway from 1993 to 2022, in a mainly screening-naive population. We will describe the incidence patterns in EOCRC compared with older age groups, stratified by subsite and morphology, to investigate differences with possible implications for prevention and treatment.

## Materials and methods

### Study design and study population

The data of all CRCs diagnosed between 1993 and 2022 were extracted from the Cancer Registry of Norway (CRN). The completeness for colon cancer is estimated to be 99.8% and for rectal cancer 99.9%.^18^ The corresponding population data were available from the National Statistics Bureau Statistics Norway.

In 1993 the coding system Seventh Revision of the International Classification of Diseases (ICD-7) was replaced with the International Classification of Diseases for Oncology (ICD-O) system, providing more detailed information on anatomical subsite and morphology.^18^ For this reason, we chose to include patients diagnosed from 1993 to 2022. In addition, the growing concerns of increasing EOCRC in recent years mandated up-to-date data. The study was approved by the Regional Committees for Medical and Health Research Ethics (REK Sør-Øst: 540745). Informed content was waived because the study was strictly registry based.

### Morphology

The CRN provided detailed information about tumor morphology according to the ICD-O system. We excluded histologic subtypes considered not relevant, such as sarcomas, melanomas, squamous cell carcinomas, and others. The remaining histologic subtypes were grouped as adenocarcinomas, mucinous and signet ring cell carcinomas (MSCs), and NENs (Supplementary Table S1, available at https://doi.org/10.1016/j.esmogo.2024.100065).

NENs are classified according to the World Health Organization (WHO) 2019 classification as neuroendocrine tumors, neuroendocrine carcinomas, or mixed neuroendocrine non-neuroendocrine neoplasms (MiNENs) based on morphological features. Morphological codes provided information on the tumor subtype (Supplementary Table S1, available at https://doi.org/10.1016/j.esmogo.2024.100065). We divided these into two groups based on known behavior: low/intermediate and high aggressiveness (Supplementary Table S2, available at https://doi.org/10.1016/j.esmogo.2024.100065). As there are large differences in the management and prognosis between these groups, we wanted to investigate which of the entities dominated the incidence of colorectal NEN.

### Primary tumor location

Tumor location was coded according to ICD-O3. Cancers in the cecum, ascending colon, hepatic flexure, and transverse colon were coded as right-sided colon cancers, while cancers in the splenic flexure, descending colon, sigmoid, and rectosigmoid junction were coded as left-sided colon cancers (Supplementary Table S3, available at https://doi.org/10.1016/j.esmogo.2024.100065). Cancers in the appendix were excluded, because of the indolent nature of most appendiceal cancers (mostly low-grade neuroendocrine tumors). These cancers were registered as malignant only from 2010 onward, which would give a false impression of a sudden rise in right-sided cancers.

### Stage

Disease stage at diagnosis was coded into three categories, consistent with stages in the Surveillance, Epidemiology and End Results Program (SEER) system. The categories were defined as (i) localized if the cancer had not metastasized to lymph nodes or other organs, (ii) regional if the cancer had metastasized to regional lymph nodes and/or has direct extension to surrounding tissues/organs, and (iii) distant if the cancer had metastasized to distant lymph nodes or other organs. The stage was coded as unknown if data could not reliably assert the extent of the disease.

### Statistical analysis

Age-standardized incidence was calculated using Stata 17 (StataCorp, College Station, TX). The rates were adjusted to the 1960 Segi world standard population^19^ and are expressed per 100 000 person-years. Incidence was stratified by age at diagnosis, grouped as 20-49 years, 50-74 years, and ≥75 years. Temporal patterns were analyzed using the Joinpoint Regression Program (Version 4.9.1.0, National Cancer Institute). Joinpoint regression analysis was used to detect statistically significant changes in time series and allows the identification of trend change points (joinpoints). The software also calculates annual percentage change (APC) and average annual percentage change (AAPC) with a 95% confidence interval (CI). Two-sided *P* values of <0.05 were considered statistically significant. In some cases, there were years with an incidence of 0 in a subgroup. To be able to perform joinpoint analyses in these subgroups, the incidence rate was manually set to 0.0001.

To examine the relationship between age groups and tumor characteristics such as morphology, we used standard chi-square tests.

## Results

There were 116 454 cases of CRC from 1993 to 2022. Of these, 6797 cases were excluded based on morphology, and 2123 cases based on an anatomical subsite (appendix). In the age group <20 years there were 11 remaining cases, and these were excluded because of the rarity of CRC in this age group. Finally, 107 523 cases were eligible for the analysis (Supplementary Figure S1, available at https://doi.org/10.1016/j.esmogo.2024.100065). The median age was 73 years. The EOCRC group (age 20-49 years) had a median age of 44 years and accounted for 5.2% of cases. In this age group, right-sided colon cancer represented 27.3% of cases, while in the oldest age group (≥75 years) right-sided colon cancer represented 45.8% (Table 1). There was a higher proportion of patients with distant metastatic disease at diagnosis in the EOCRC group, 28.6% as compared with 18.2% in the age group ≥75 years, and 23.1% in the age group 50-74 years (*P* < 0.001).

**Table 1:** Distribution of sex, primary tumor location, stage, and morphology across age groups for all colorectal cancer cases diagnosed 1993-2022

	Age 20-49 years (*n* = 5591), *n* (%)	Age 50-74 years (*n* = 55 358), *n* (%)	Age ≥75 years (*n* = 46 574), *n* (%)	Total (*n* = 107 523), *n* (%)
**Sex**				
Male	2791 (49.9)	30 525 (55.1)	21 736 (46.7)	55 052 (51.2)
Female	2800 (50.1)	24 833 (44.9)	24 838 (53.3)	52 471 (48.8)
**Primary tumor location**				
Right-sided colon	1525 (27.3)	18 684 (33.8)	21 312 (45.8)	41 521 (38.6)
Left-sided colon	1888 (33.8)	17 885 (32.3)	12 935 (27.8)	32 708 (30.4)
Rectum	2078 (37.2)	17 851 (32.3)	11 391 (24.5)	31 320 (29.1)
NOS	100 (1.8)	938 (1.7)	936 (2.0)	1974 (1.8)
**Stage**				
Localized	1028 (18.4)	11 896 (21.5)	10 208 (21.9)	23 132 (21.5)
Regional	2652 (47.4)	27 959 (50.5)	24 484 (52.6)	55 095 (51.2)
Distant	1597 (28.6)	12 807 (23.1)	8460 (18.2)	22 864 (21.3)
Unknown	314 (5.6)	2696 (4.9)	3422 (7.4)	6432 (6.0)
**Morphology**				
Adenocarcinoma	4791 (85.7)	49 490 (89.4)	41 296 (88.7)	95 577 (88.9)
MSC	553 (9.9)	5015 (9.1)	4875 (10.5)	10 443 (9.7)
NEN	247 (4.4)	853 (1.5)	403 (0.9)	1503 (1.4)

MSC, mucinous and signet ring cell carcinoma; NEN, neuroendocrine neoplasm; NOS, not otherwise specified.

In an analysis of all age groups, 88.9% were adenocarcinomas, 9.7% were MSCs, and 1.4% NENs. The proportion of neuroendocrine cases was significantly higher (*P* < 0.001) in the EOCRC age group (4.4%) compared with the 50-74-year age group (1.5%) and those ≥75 years of age (0.9%). Of the MSCs, the majority of cases were mucinous adenocarcinomas (94.2%) and a small proportion were signet ring cell carcinomas (5.6%). The remaining cases (0.2%) were mucinous cystadenocarcinomas.

### Incidence patterns in the age group 20-49 years

In the youngest age group, the age-adjusted incidence rate increased by 66%, from 6.8/100 000 in 1993 to 11.2/100 000 in 2022 (AAPC 1.3; 95% CI 0.9-1.6); further details are given in Supplementary Table S4, available at https://doi.org/10.1016/j.esmogo.2024.100065. The increasing trend was continuous throughout the period, with no sign of stabilization or decline (Supplementary Figure S2A, available at https://doi.org/10.1016/j.esmogo.2024.100065).

**Figure 1:**
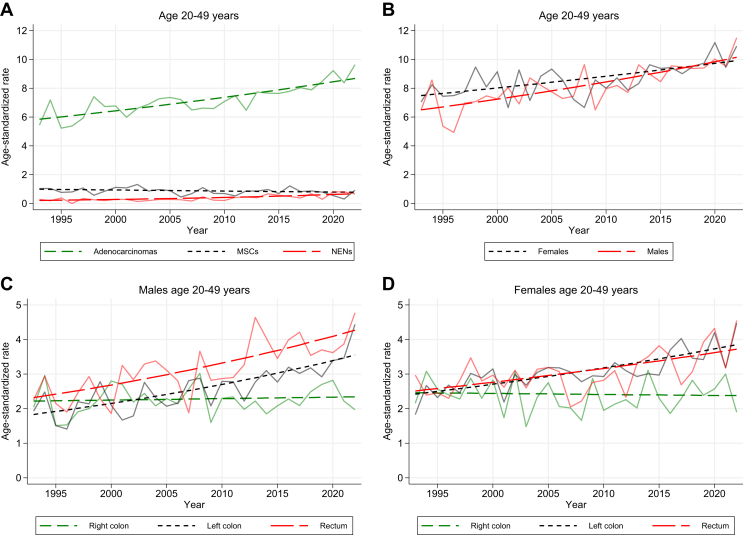
**Age-standardized incidence rates (ASRs) per 100 000 in the age group 20-49 years from 1993 to 2022.** (A) Stratified by morphology. (B) Stratified by sex. (C) Males, stratified by primary tumor location. (D) Females, stratified by primary tumor location. MSC, mucinous and signet ring cell carcinoma; NEN, neuroendocrine neoplasm.

When stratifying based on morphology, adenocarcinomas increased by 77%, from 5.4 to 9.6/100 000 (AAPC 1.4; 95% CI 1.0-1.7, Figure 1A). Comprising the majority of cases, they were the primary contributors to the overall increase. NENs increased sharply, but because of their infrequency, the impact on the overall incidence was minimal (AAPC 4.1; 95% CI 2.7-5.6). In the 3 most recent years (2020-2022), NENs accounted for ∼7% of cases in this age group compared with 4% in 1993-1995. The proportion of NENs was highest in rectal cancer, accounting for 9% of cases.

Males had a lower incidence compared with females at the beginning of the period, but a sharper increase in incidence (AAPC 1.5 for males versus 1.0 for females; Figure 1B). Concerning primary tumor location, the increase in incidence was mainly caused by left-sided colon and rectal cancer (AAPC 2.0; 95% CI 1.5-2.4 and AAPC 1.8; 95% CI 1.2-2.3), while right-sided cancer had a stable incidence (AAPC 0.1; 95% CI –0.5 to 0.6; Supplementary Figure S3A, available at https://doi.org/10.1016/j.esmogo.2024.100065). The sharpest increase was seen for left-sided colon and rectal cancers in males (AAPC 2.3; 95% CI 1.6-3.0 and 2.1; 95% CI 1.4-2.8; Figure 1C). For females, the incidence of left-sided colon and rectal cancers increased as well, but at a slightly lower pace (AAPC 1.6; 95% CI 1.1-2.1 and AAPC 1.4; 95% CI 0.7-2.0, Figure 1D).

### Incidence patterns in the age group 50-74 years

**Figure 2:**
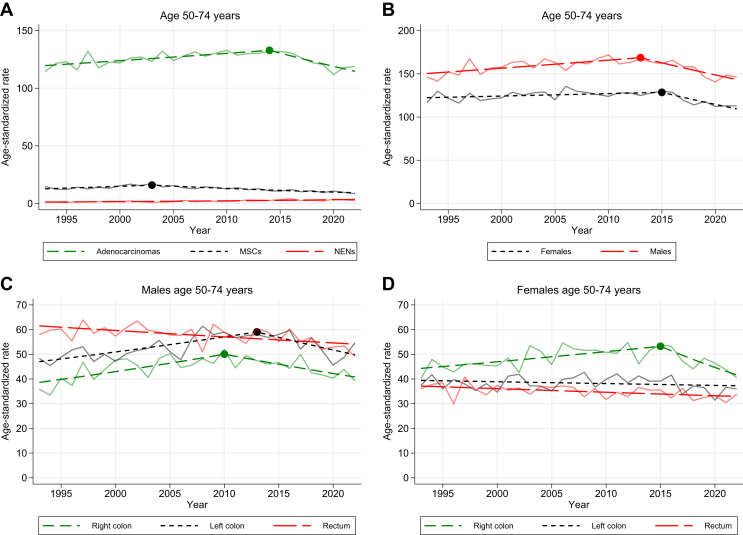
**Age-standardized incidence rates (ASRs) per 100 000 in the age group 50-74 years from 1993 to 2022.** (A) Stratified by morphology. (B) Stratified by sex. (C) Males, stratified by primary tumor location. (D) Females, stratified by primary tumor location. MSC, mucinous and signet ring cell carcinoma; NEN, neuroendocrine neoplasm.

In the age group 50-74 years the overall incidence rate was similar in 1993 as in 2022, ∼130/100 000. Rectal cancer had a modest decrease throughout the period (AAPC –0.4; 95% CI –0.6 to –0.2). Right-sided cancer initially increased with an APC of 1.5 up to 2010; however, from 2010 onward, it decreased with an APC of –1.7 (Supplementary Figure S3B, available at https://doi.org/10.1016/j.esmogo.2024.100065). Left-sided cancer had a similar pattern as right-sided cancer overall but differed between the sexes. For males, left-sided cancer increased with an APC of 1.1 up until 2013 and then decreased with an APC of –1.9 (Figure 2C). For females, the incidence of left-sided cancer was stable with no joinpoints (AAPC –0.2; 95% CI –0.5 to 0.1; Figure 2D).

### Incidence patterns in the age group ≥75 years

**Figure 3:**
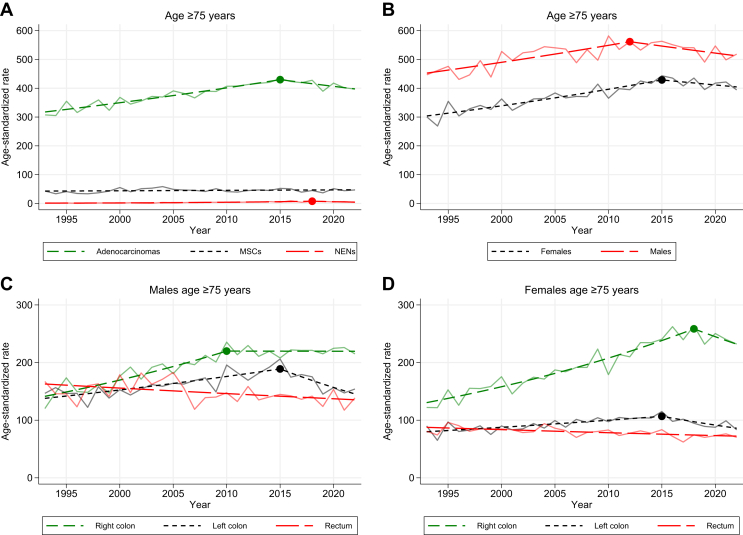
**Age-standardized incidence rates (ASRs) per 100 000 in the age group ≥75 years from 1993 to 2022**. (A) Stratified by morphology. (B) Stratified by sex. (C) Males, stratified by primary tumor location. (D) Females, stratified by primary tumor location. MSC, mucinous and signet ring cell carcinoma; NEN, neuroendocrine neoplasm.

In the oldest age group, there was a 27% increase in incidence rate from 352/100 000 in 1993 to 448/100 000 in 2022. An increase was observed from 1993 to 2015 (APC 1.4), while the incidence decreased from 2015 to 2022 (APC –1.1, Supplementary Figure S2C, available at https://doi.org/10.1016/j.esmogo.2024.100065). The increase was mainly driven by right-sided cancer where the incidence rate increased by 85%, from 122/100 000 in 1993 to 224/100 000 in 2022. This was especially pronounced in females (Figure 3D). Right-sided cancer in the older age group increased with an APC of 2.5 up until 2016, followed by a slight decrease with an APC of –0.9 (Supplementary Figure S3C, available at https://doi.org/10.1016/j.esmogo.2024.100065).

Males had a higher incidence of CRC, but females had a steeper increase in the incidence, especially for right-sided cancer (females AAPC 2.0 versus males AAPC 1.5; Figure 3C and D). Right-sided cancer in females increased up until 2018 (APC 2.8), but then decreased from 2018 to 2022 (APC –2.7). The incidence of right-sided cancer in females surpassed that of males in 2015. Right-sided cancer in males increased from 1993 to 2010 (APC 2.6) and was stable from 2010 to 2022 (APC 0.0; Figure 3C).

### Incidence patterns in morphological subgroups

#### Adenocarcinomas

**Figure 4:**
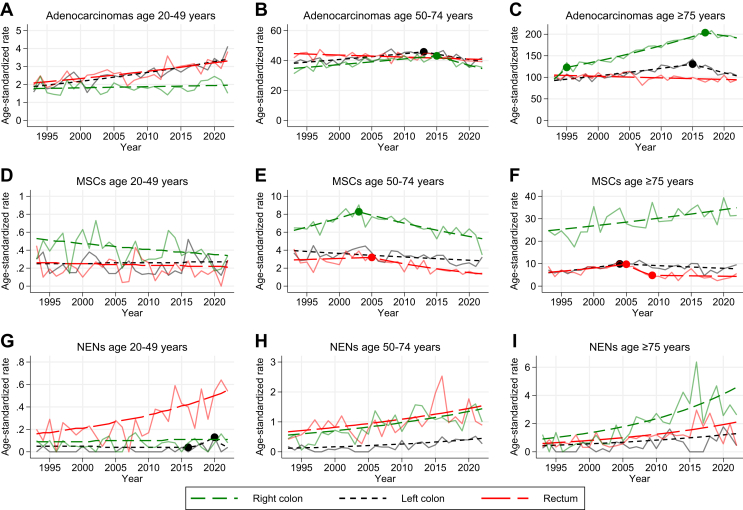
**Age-standardized incidence rate (ASRs) per 100 000 from 1993 to 2022 for adenocarcinomas, MSCs, and NENs in different age groups, by primary tumor location**. (A-C) Adenocarcinomas, stratified by primary tumor location. (D-F) Mucinous and signet ring cell carcinomas, stratified by primary tumor location. (G-I) Neuroendocrine neoplasms, stratified by primary tumor location. MSC, mucinous and signet ring cell carcinoma; NEN, neuroendocrine neoplasm.

As the nonmucinous adenocarcinomas account for the majority of cases, the distribution of incidence across age groups and primary tumor locations is similar to that of the overall incidence. The increase in the EOCRC group was somewhat more profound when including only nonmucinous adenocarcinomas, mainly because of left-sided colon and rectal cancer (Figure 4A). For the age group 50-74 years there were no major trends, but a tendency toward stabilization or decline in recent years (Figure 4B). For the oldest age group, we observed the same trend as that for the overall incidence, with a decline in right-sided cancer after 2017 (Figure 4C).

#### Mucinous and signet ring cell carcinomas

MSCs had an overall stable incidence (AAPC −0.6; 95% CI –1.3 to 0.2). This morphological subtype is most common in the right colon, particularly in those aged ≥50 years (Figure 4E and F). For the youngest age group, the overall incidence was low and with a tendency toward decline, especially right-sided colon cancer (AAPC –1.5; 95% CI –2.9 to –0.3; Figure 4D).

#### Neuroendocrine neoplasms

For NENs, the incidence was low, but an increase was seen in all age groups (Figure 4G-I). In the age group 20-49 years, the incidence rate increased by 131% from 0.29/100 000 in 1993 to 0.67/100 000 in 2022 (AAPC 4.1; 95% CI 2.7-5.6). The increase was almost exclusively caused by a rise in rectal NENs (AAPC 4.3; 95% CI 2.8-5.9; Figure 4G). The incidence of NENs located in the colon was extremely low throughout the period in this age group.

For the age group 50-74 years the most pronounced increase was in right-sided and rectal NEN (Figure 4H).

In the older age group (≥75 years), there was an overall increase for all sites and both sexes (AAPC 4.9; 95% CI 2.0-7.9). However, there was a trend-shift in 2018; the APC before 2018 was 7.8, and after it was –11.7 (Figure 3A).

#### Distribution of highly aggressive versus low-/intermediate-aggressive NEN

There was a larger proportion of highly aggressive NENs in the older age groups and in the colon (Table 2). In the age group 20-49 years, the increase in NEN incidence was mainly attributed to the low-/intermediate-aggressive NENs located in the rectum. In the age group 50-74 years, there was an increase of both high and low-/intermediate-aggressive NENs, but low-/intermediate-aggressive NENs had a slightly higher incidence. In the age group ≥75 years, the proportion of highly aggressive NENs was higher, and they were the main drivers of the increased incidence in this age group (Supplementary Figure S4, available at https://doi.org/10.1016/j.esmogo.2024.100065).

**Table 2:** Distribution of aggressiveness of neuroendocrine neoplasms across age groups and primary tumor location

	Aggressiveness			
	Low/intermediate, *n* (%)	High, *n* (%)	Total	*P* value∗
**Age group (years), *n* (%)**				<0.001
20-49	193 (78.1)	54 (21.9)	247	
50-74	447 (52.4)	406 (47.6)	852	
≥75	148 (36.7)	255 (63.3)	401	
**Primary tumor location, *n* (%)**				<0.001
Right-sided colon	231 (37.0)	393 (63.0)	622	
Left-sided colon	46 (30.3)	106 (69.7)	152	
Rectum	479 (71.8)	188 (28.2)	666	
NOS	32 (53.3)	28 (46.7)	60	
Total	788 (52.4)	715 (47.6)	1503	

NOS, not otherwise specified.

∗ *P* value from Pearson’s chi-square test.

## Discussion

In this analysis of incidence trends in Norway over the past three decades, we found a continuing increasing incidence of EOCRC mainly because of left-sided colon and rectal cancers. In the older age group, right-sided colon cancer increased until recently, while the incidence of rectal cancer has either stabilized/slightly decreased. There was no national screening program in Norway during the study period, so the differences between incidence patterns in early- and late-onset cancer are most likely not solely attributable to population-based screening.

CRC screening has probably led to a reduction of LOCRC incidence in most countries with established screening programs, despite modest results in randomized trials.^3^^,^^20^ By contrast, our study found an increasing incidence in the older population up until recent years. The most recent decrease could potentially be explained by increased endoscopic activity as a result of a pilot screening program that was initiated in 2012.^21^ However, the program only affected a minor part of the population and the impact on national incidence data is uncertain. We carried out a supplementary analysis of incidence stratified by county, comparing counties participating in the pilot program with those that did not (data not shown). The analysis demonstrated a significant trend shift in CRC incidence in both groups from the mid-2010s, suggesting a multifactorial explanation for the most recent decline. In addition to the impact of the pilot program and/or raised national awareness with regard to screening, other possible factors could be changes in lifestyle and diets across time.

In most screening programs the population <50 years of age is not included, and in Norway, the current screening starts at age 55 years.^3^ However, the American Cancer Society has recommended screening for the average-risk population to start at the age of 45 years, because of the increasing incidence of EOCRC.^22^ Although the incidence has increased, the absolute risk remains low in this age group. Identifying risk groups eligible for screening would be a feasible method of prevention, but so far, family history is the only risk factor that leads to a screening recommendation in the younger population.^23^

Treatment guidelines for CRC are largely based on research carried out on patients primarily in the age group 50-74 years. The problem of extrapolating results between age groups is well-known when treating older patients, but this may also pose a challenge for EOCRC. The Delphi Initiative for Early-Onset Colorectal Cancer (DIRECt) recently published guidelines for the management of EOCRC. The most important recommendation is germline genetic testing of all patients with EOCRC. Treatment recommendations are in principle the same as for LOCRC.^23^ The International Duration Evaluation of Adjuvant Chemotherapy (IDEA) Consortium reported that patients with low-risk stage III (T1-3N1) colon cancer could safely be treated with 3 months of adjuvant capecitabine and oxaliplatin (CAPOX) instead of 6 months.^24^ However, subgroup analyses indicated that in the EOCRC population, 3 months of adjuvant treatment resulted in significantly lower disease-free survival in the low-risk stage III group, suggesting that this treatment duration may not be sufficient for EOCRC.^25^ Given the low incidence of EOCRC, clinical trials for this patient group alone would be hard to conduct, but larger trials and meta-analyses should present age-stratified results when feasible.

The higher frequency of metastatic disease at diagnosis in EOCRC compared with older patients (Table 1) is in line with findings from other studies,^6^^,^^8^ and may suggest age-specific differences in tumor biology and prognosis. This tendency seen in our material cannot be explained by the lack of screening in EOCRC, as there were no national screening programs for the older age groups in the study period. A delayed diagnosis as a result of a lack of awareness among physicians and the general population could perhaps explain this, but more likely it is a combination of factors.

NENs are rapidly increasing in all age groups. In the age group 20-49 years, rectal NENs are somewhat affecting the overall EOCRC incidence, comprising 9% of rectal cancers. The analysis of the incidence of EOCRC stratified based on morphology and subsite in the United States by Montminy et al.^17^ found a similar increase in rectal NENs. The proportion of NENs seemed to be higher than in our study, reporting 4%-20% of all CRC cases, and 8%-34% of rectal cancer, depending on age group and calendar year.

There are no identified genomic drivers in sporadic EOCRC that is substantially different from LOCRC.^26^ However, the incidence patterns based on tumor location indicate that the carcinogenic pathway and etiology of these cancers differ, and subsite-specific risk factors for CRC have been identified.^27^^,^^28^

The rising incidence of EOCRC is described in several high-income countries, despite the fact that some countries observe a decline (e.g. Italy and Austria). We found an overall annual increase in incidence of 1.3%, comparable with Sweden (1.7%), Denmark (1.0%), and Germany (1.3%).^29^ The increase in early-onset disease is also observed in other gastrointestinal malignancies, including gastric, pancreatic, and biliary cancers.^26^ There are several risk factors associated with EOCRC, including Western diet, obesity, sedentary lifestyle, and non-alcoholic fatty liver disease.^30^ The use of antibiotics in childhood or adolescence has also been linked to EOCRC.^31^ The reasons for the rising incidence of EOCRC are likely multifactorial, and genetics in combination with carcinogenic exposure in childhood and adolescence are plausible explanations that need to be explored further.

This study has some limitations. The absence of detailed data on lifestyle factors and other demographic variables in the CRN limits the possibility of studying potential risk and etiological factors. Linking EOCRC incidence data from the CRN to other registries with more extensive demographic information could be an attainable method for exploring this in future studies. In addition, changes in coding and routine diagnostic practices over time are potential biases. This is especially significant for the neuroendocrine morphological subgroups. We chose to exclude appendiceal cancers because the most indolent lesions were previously coded as benign, leading to a false sudden increase ∼2010. To our knowledge, this change in coding practice does not apply to other colorectal NENs. The increase in NEN incidence may partially be explained by more cancers being diagnosed as NENs by the introduction of new diagnostic subgroups such as MANEC (mixed adenoneuroendocrine carcinoma) or later MiNEN, or by increased use of immunohistochemical markers in routine practice. Increased detection using radiological or endoscopic procedures could also pose a bias, especially for low-grade NENs that are often incidental findings. Despite these limitations, the CRN provides data that are >99% complete,^18^ enabling us to give a comprehensive insight into incidence trends of this nationwide cohort.

In summary, there is an increasing trend of EOCRC incidence in Norway, caused by left-sided colon and rectal cancers. This is in line with findings from other epidemiological studies and necessitates a deeper understanding of the underlying mechanisms for EOCRC to take adequate actions for prevention and treatment strategies. In the older age group, there has been a trend of increasing incidence of mainly colon cancer up until recently, with a trend shift noted around the end of the study period. Only time will show if the most recent decline is sustained with the expansion from a limited pilot to a national screening program.
