# Supplementary material for: Early-onset colorectal cancer incidence in Norway: a national registry-based study (1993-2022) analyzing subsite and morphology trends
Source: ESMO Gastrointest Oncol. 2024 Aug 2;7:100065. doi: 10.1016/j.esmogo.2024.100065 (PMC12836505; doi:10.1016/j.esmogo.2024.100065)
Supplement: Supplementary Tables and Figures [file mmc1.pdf]

Supplementary Material

TITLE: Incidence patterns of colorectal cancer in Norway 1993–2022 by age, subsite and morphology.

Page 1	Content
Page 2	Table 1: The International Classification of Diseases for Oncology, Third Edition (ICD-O3) morphology codes, grouped in three categories
Page 3	Table 2: Division of morphological subgroups of neuroendocrine neoplasms and frequency of these.
	Table 3: The international classification of Diseases for Oncology, Third Edition (ICD-O3) codes defining primary tumor location.
Page 4	Table 4: Age-standardized incidence rates per 100 000 person-years and corresponding APC values
Page 5:	Figure 1: CONSORT diagram
Page 6:	Figure 2: Age-standardized incidence rates per 100 000 from 1993–2022 by age groups.
Page 7:	Figure 3: Age-standardized incidence rates per 100 000 by age groups and primary tumor location.
Page 8:	Figure 4: Age-standardized incidence rates for neuroendocrine neoplasms, stratified for high and low/intermediate aggressiveness.

Table 1. The International Classification of Diseases for Oncology, Third Edition (ICD-O3) morphology codes, grouped in three categories.

**Morphology group**	**Morphology codes (ICD-O3)**	**Description**
Adenocarcinomas	81403	Adenocarcinoma, NOS
	81443	Adenocarcinoma, intestinal type
	81453	Adenocarcinoma, diffuse type
	82013	Cribriform carcinoma, NOS
	82103	Adenocarcinoma in adenomatous polyp
	82113	Tubular adenocarcinoma
	82133	Serrated adenocarcinoma
	82433	Goblet cell adenocarcinoma
	82553	Adenocarcinoma with mixed subtypes
	82603	Papillary adenocarcinoma, NOS
	82613	Adenocarcinoma in villous adenoma
	82623	Villous adenocarcinoma
	82633	Adenocarcinoma in tubulovillous adenoma
	82653	Micropapillary carcinoma, NOS
	83103	Clear cell adenocarcinoma, NOS
Mucinous and signet-ring cell carcinomas	84703	Mucinous cystadenocarcinoma, NOS
	84803	Mucinous adenocarcinoma
	84813	Mucin-producing adenocarcinoma
	84903	Signet ring cell carcinoma
Neuroendocrine neoplasms	80133	Large cell neuroendocrine carcinoma
	80413	Small cell carcinoma
	81543	Mixed neuroendocrine non-neuroendocrine neoplasm (MiNEN)
	82403	Neuroendocrine tumor
	82413	Enterochromaffin cell carcinoid
	82493	Neuroendocrine tumor, grade 2
	82463	Neuroendocrine carcinoma
	82443	Mixed adenoneuroendocrine carcinoma
	82453	Adenocarcinoid tumor

NOS; Not otherwise specified

Table 2. Division of morphological subgroups of neuroendocrine neoplasms and frequency of these.

**Low/intermediate aggressiveness**	**High aggressiveness**	**Number of cases**	**%**
	80133 Large cell neuroendocrine carcinoma	76	5.6
	80413 Small cell carcinoma	73	4.9
	82463 Neuroendocrine carcinoma	465	30.9
	82443 Mixed adenoneuroendocrine carcinoma	98	6.5
	81543 Mixed neuroendocrine non-neuroendocrine neoplasm (MiNEN)	3	0.2
82453 Adenocarcinoid tumor		14	0.9
82403 Neuroendocrine tumor		691	46.0
82413 Enterochromaffin cell carcinoid		1	0.1
82493 Neuroendocrine tumor, grade 2		82	5.5
Total		1503	100

Table 3. The international classification of Diseases for Oncology, Third Edition (ICD-O3) codes defining primary tumor location.

**Primary tumor location**	**ICD-O3 Code and description**
Right sided colon cancer	C18.0 Caecum<br>C18.2 Ascending colon<br>C18.3 Hepatic flexure of colon<br>C18.4 Transverse colon
Left Sided colon cancer	C18.5 Splenic flexure of colon<br>C18.6 Descending colon<br>C18.7 Sigmoid colon<br>C19.9 Rectosigmoid junction
Rectal cancer	C20.9 Rectum, NOS
Unknown	C18.8 Overlapping lesion of colon<br>C18.9 Colon, NOS
Excluded	C18.1 Appendix

NOS; not otherwise specified

Table 4. Age-standardized incidence rates per 100 000 person-years and corresponding APC values

	1993			2022			Trend 1 APC (%)	Trend 2 APC (%)	Joinpoint	AAPC (%)	AAPC 95% CI
	Cases	ASR	95% CI	Cases	ASR	95% CI					
**All age groups**	2720	55.4	53.1, 57.8	4516	61.3	59.3, 63.3	1.2<br>0.4	-1.3	2002<br>2015	0.2	-0.0, 0.5
RCC	873	16.9	15.7, 18.2	1783	20.8	19.7, 21.9	2.7<br>0.8	2.1	2000<br>2016	0.7*	0.2, 1.1
LCC	853	17.6	16.3, 19.0	1383	20.4	19.2, 21.6	1.0	1.5	2014	0.3	-0.1
Rectum	926	19.4	18.0, 20.8	1255	18.9	17.8, 20.1	-0.2		-	-0.2	-0.3, 0.0
**Age 20–49**	134	6.8	5.7, 8.0	269	11.2	9.9, 12.6	1.3		-	1.3*	0.9, 1.6
RCC	42	2.1	1.5, 2.8	46	1.9	1.4, 2.6	0.1		-	0.1	-0.5, 0.6
LCC	38	1.9	1.3, 2.6	107	4.5	3.6, 5.4	2.0		-	2.0*	1.5, 2.4
Rectum	52	2.6	1.9, 3.4	112	4.7	3.8, 5.6	1.8		-	1.8*	1.2, 2.3
**Adenocarcinoma Age 20–49**	109	5.4	4.5, 6.6	231	9.6	8.4, 11.0	1.4		-	1.4*	1.0, 1.7
RCC	34	1.7	1.2, 2.4	36	1.5	1.1, 2.1	0.3		-	0.3	-0.3, 1.0
LCC	32	1.6	1.1, 2.2	99	4.1	3.4, 5.0	2.1		-	2.1*	1.6, 2.6
Rectum	39	2.0	1.4, 2.7	92	3.8	3.1, 4.7	1.6		-	1.6	1.0, 2.2
**MSC Age 20–49**	21	1.0	0.7, 1.6	22	0.9	0.6, 1.4	-0.8		-	-0.8	-1.9, 0.3
RCC	6	0.3	0.1, 0.7	8	0.3	0.2, 0,7	-1.5		-	-1,5	-2.6, 1.3
LCC	6	0.3	0.1, 0.6	7	0.3	0.1, 0.6	0.3		-	0.3	-1.4, 2.1
Rectum	9	0.4	0.2, 0.9	7	0.3	0.1 0.6	-0.7		-	-0.7	-2.6, 0.8
**NEN Age 20–49**	6	0.3	0.1, 0.6	16	0.7	0.4, 1.1	4.1		-	4.1*	2.7, 5.6
RCC	2	0.1	0.1, 0.1	2	0.1	0.0, 0.3	0.9		-	0.9	-0.8, 2.7
LCC	0	0.0	0.0, 0.2	1	0.0	0.0, 0.2	3.4		-	3.4*	1.4, 5.4
Rectum	4	0.2	0.1, 0.5	13	0.5	0.3, 0.9	4.3		-	4.3*	2.7, 5.9
**Age 50–74**	1474	130.4	123.5, 137.5	2225	129.7	124.2, 135.3	0.4	-2.0	2014	-0.2*	-0.5, -0.0
RCC	446	38.2	34.6, 42.1	718	40.0	37.0, 43.1	1.4	-1.7	2010	0.1	-0.2, 0.5
LCC	468	42.4	38.5, 46.6	762	45.3	42.1, 48.7	0.8	-1.8	2013	0.0	-0.5, 0.5
Rectum	526	46.4	42.3, 50.7	695	41.5	38.4, 44.8	-0.4		-	-0.4*	-0.6, -0.2
**Age ≥75**	1110	352.4	331.8, 374.0	2022	448.1	428.8, 468.2	1.4	-1.1	2015	0.8	0.4, 1.1
RCC	385	121.3	109.4, 134.2	1019	224.6	211.0, 238.9	2.5	-0.9	2016	1.8*	1.3, 2.2
LCC	347	109.6	98.2, 121.9	514	114.6	104.7, 124.7	1.5	-3.2	2015	0.4	-0.1, 0.9
Rectum	348	111.6	100.1, 124.1	448	99.9	91.0, 109.9	-0.4		-	-0.4*	-0.7, -0.1

ASR: age-standardized incidence rate, APC: Annual percentage change, AAPC: Average annual percentage change; RCC: right sided cancer, LCC: Left-sided cancer, MSC: Mucinous or signet-ring cell carcinoma, NEN: Neuroendocrine neoplasms.
*Statistically significantly different from zero, p<0.05.

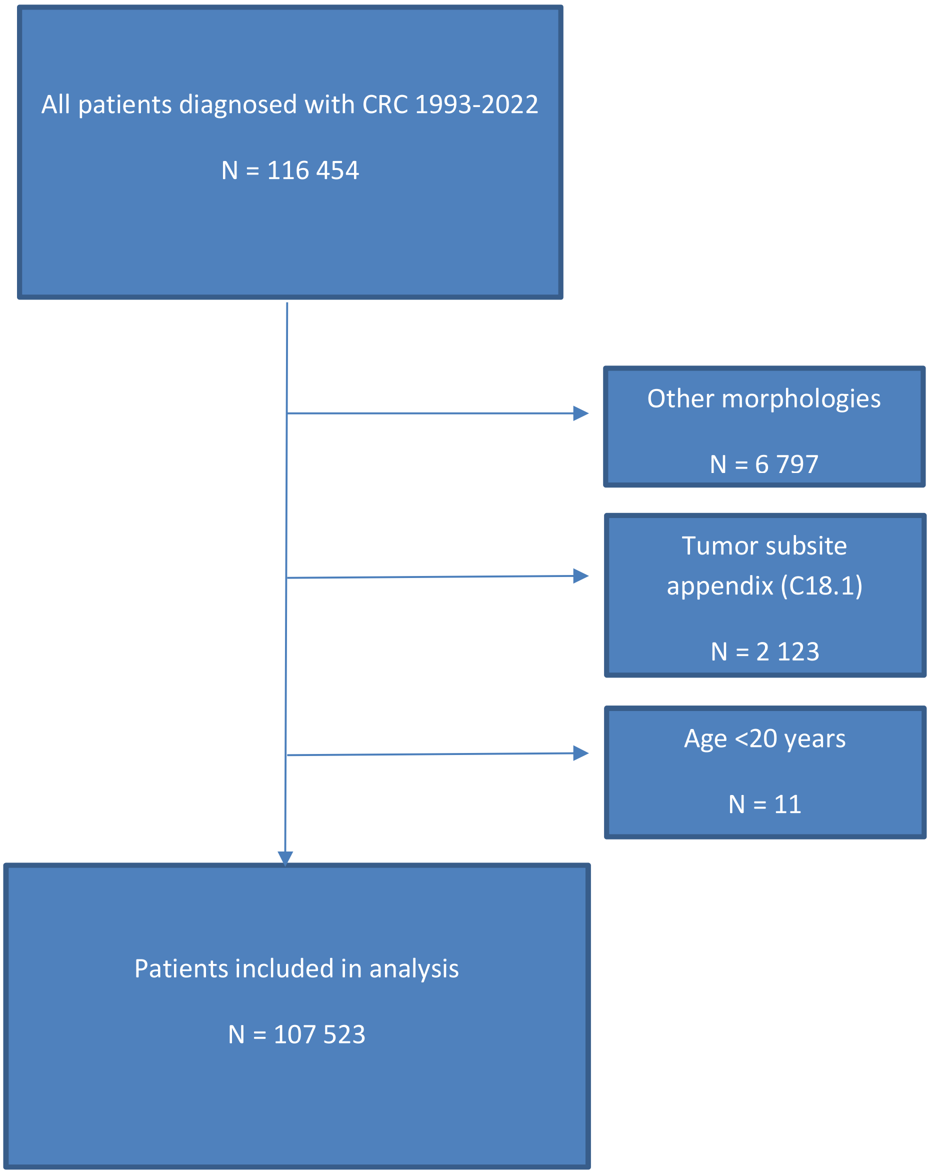

Figure 1: CONSORT diagram

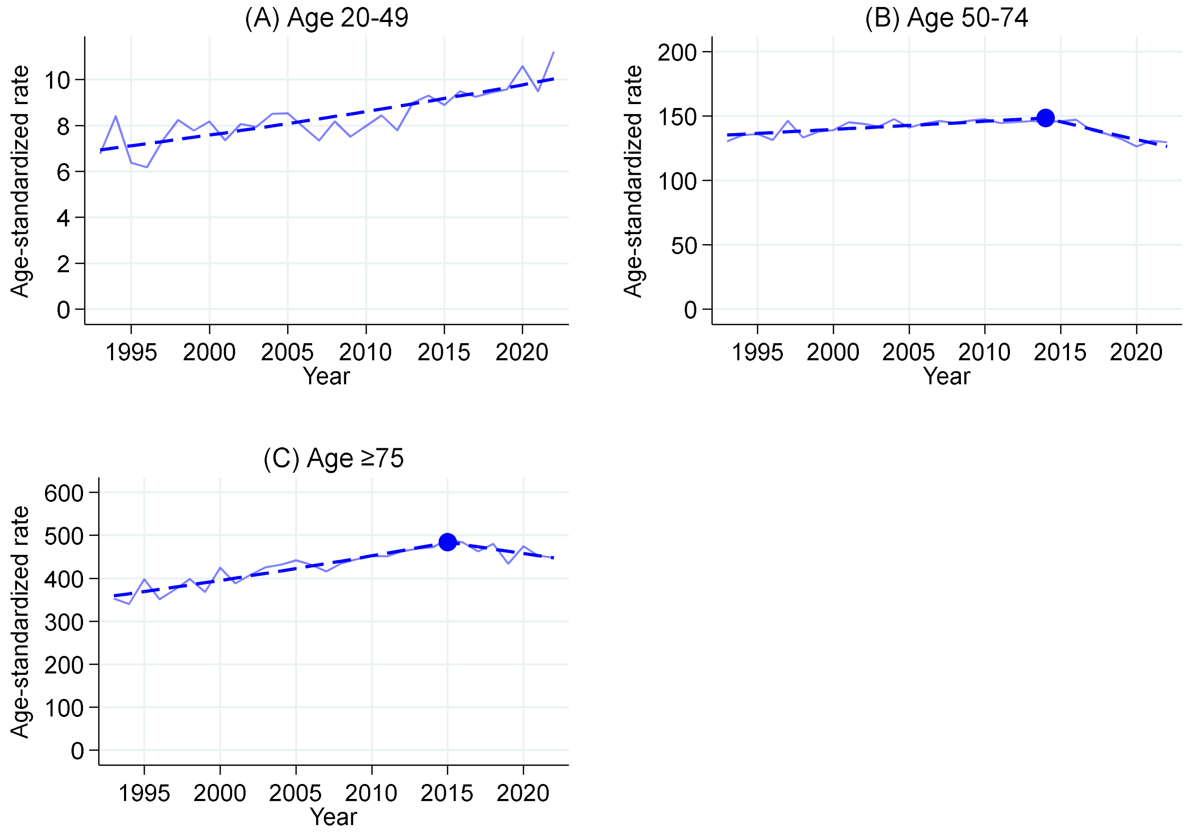

Figure 2: Age-standardized incidence rates per 100 000 from 1993-2022 of all patients in (A) age 20–49 years, (B) age 50–74 years and (C) age 75 years or older.

Dashed line: modelled incidence rate from joinpoint regression analysis.

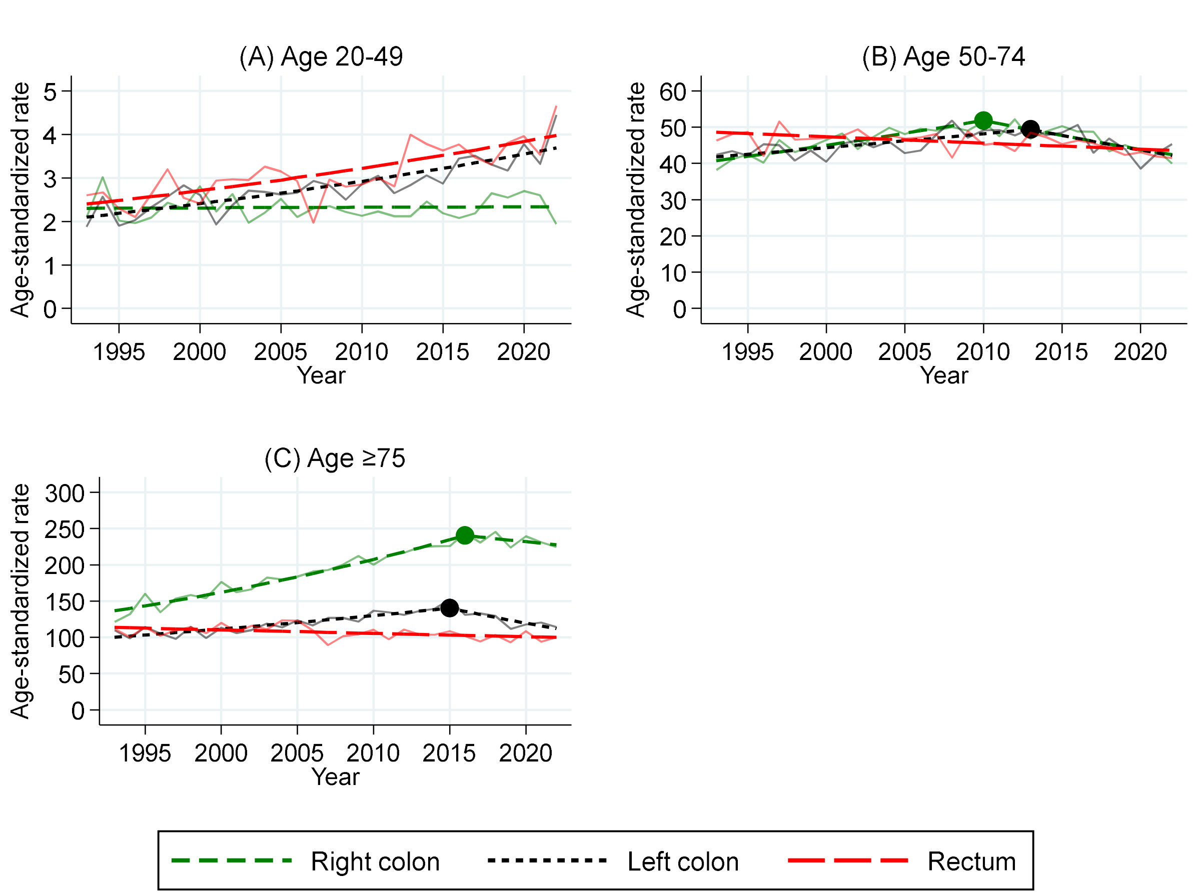

Figure 3: Age-standardized incidence rates per 100 000 from 1993 to 2022 stratified by primary tumor location. (A) Age 20–49 years, (B) age 50–74 years and (C) age 75 years or older.

Dashed line: modelled incidence rate from joinpoint regression analysis.

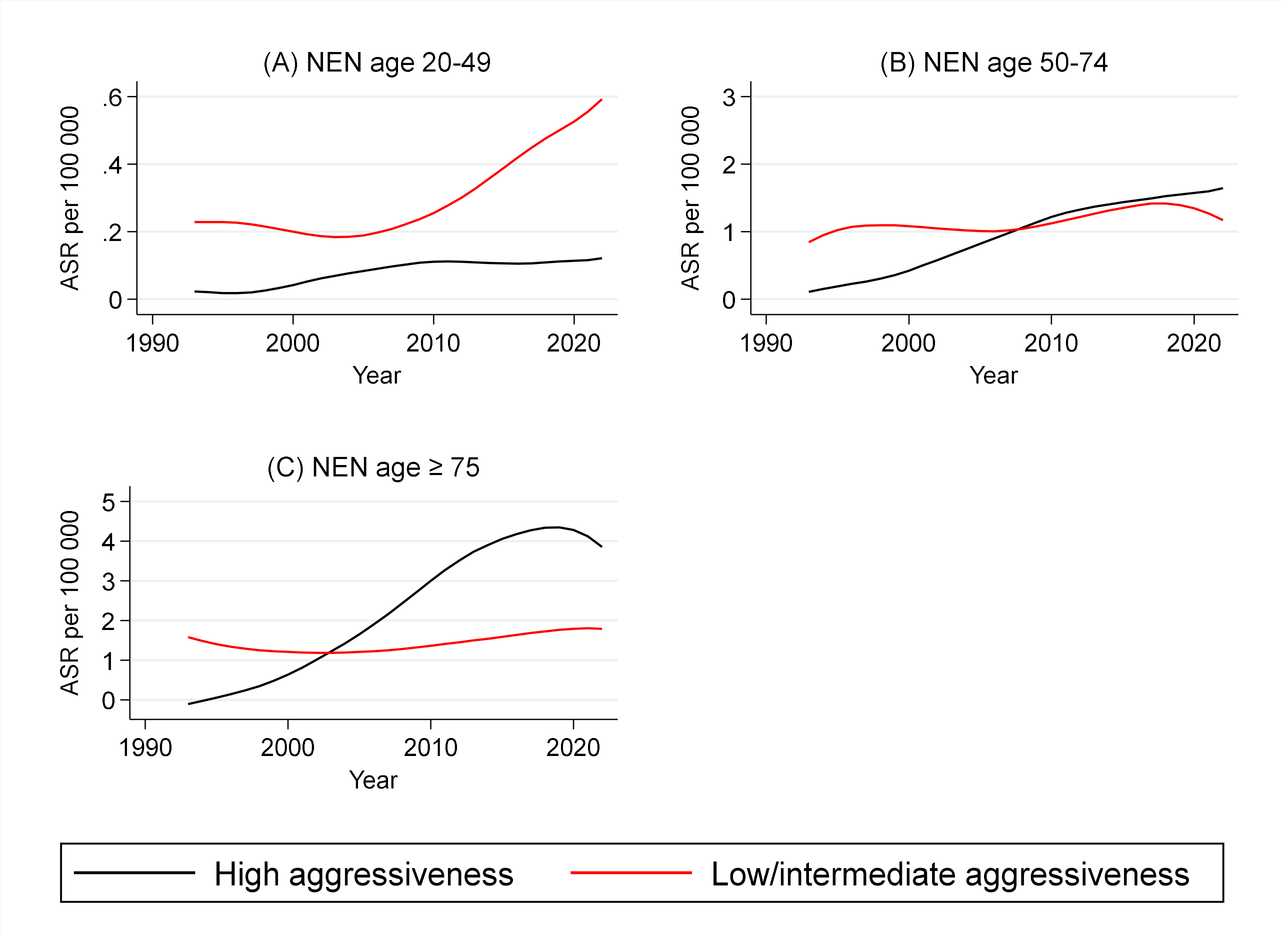

Figure 4: Age-standardized incidence rates for neuroendocrine neoplasms, stratified for high and low/intermediate aggressiveness, with smoothed lines. (A) Age 20–49, (B) age 50–74, (C) age 75 years or older.
